# A novel prognostic nomogram for colorectal cancer liver metastasis patients with recurrence after hepatectomy

**DOI:** 10.1002/cam4.3697

**Published:** 2021-02-04

**Authors:** Jie‐ying Liang, Hao‐cheng Lin, Jingwen Liu, De‐shen Wang, Yun‐fei Yuan, Bin‐kui Li, Yun Zheng, Xiao‐jun Wu, Gong Chen, Feng‐hua Wang, Zhi‐qiang Wang, Zhi‐zhong Pan, De‐sen Wan, Rui‐hua Xu, Yu‐hong Li

**Affiliations:** ^1^ State Key Laboratory of Oncology in South China Sun Yat‐sen University Cancer Center Collaborative Innovation Center for Cancer Medicine Guangzhou People's Republic of China; ^2^ Department of Medical Oncology Sun Yat‐sen University Cancer Center Guangzhou People's Republic of China; ^3^ Zhongshan School of Medicine Sun Yat‐sen University Guangzhou China; ^4^ Department of Hepatobiliary Surgery Sun Yat‐sen University Cancer Center Guangzhou People's Republic of China; ^5^ Department of Colorectal Surgery Sun Yat‐sen University Cancer Center Guangzhou People's Republic of China

**Keywords:** colorectal cancer liver metastasis, hepatectomy, nomogram, post‐recurrence survival, prognostic factors

## Abstract

**Purpose:**

We aimed to construct a nomogram to predict personalized post‐recurrence survival (PRS) among colorectal cancer liver metastasis (CRLM) patients with post‐hepatectomy recurrence.

**Methods:**

Colorectal cancer liver metastasis patients who received initial hepatectomy and had subsequent recurrence between 2001 and 2019 in Sun Yat‐sen University Cancer Center from China were included in the study. Patients were randomly assigned to a training cohort and a validation cohort on a ratio of 2:1. Univariable analysis was first employed to select potential predictive factors for PRS. Then, the multivariable Cox regression model was applied to recognize independent prognostic factors. According to the model, a nomogram to predict PRS was established. The nomogram's predictive capacity was further assessed utilizing concordance index (C‐index) values, calibration plots, and Kaplan–Meier curves.

**Results:**

About 376 patients were finally enrolled, with a 3‐year PRS rate of 37.3% and a 5‐year PRS rate of 24.6%. The following five independent predictors for PRS were determined to construct the nomogram: the largest size of liver metastases at initial hepatectomy, relapse‐free survival, CEA level at recurrence, recurrent sites, and treatment for recurrence. The nomogram displayed fairly good discrimination and calibration. The C‐index value was 0.742 for the training cohort and 0.773 for the validation cohort. Patients were grouped into three risk groups very well by the nomogram, with 5‐year PRS rates of 45.2%, 23.3%, and 9.0%, respectively (*p* < 0.001) in the training cohort and 36.0%, 9.2%, and 4.6%, respectively (*p* < 0.001) in the validation cohort.

**Conclusion:**

A novel nomogram was built and validated to enable the prediction of personal PRS in CRLM patients with post‐hepatectomy recurrence. The nomogram may help physicians in decision making.

## Graphical abstract

Independent prognostic factors for post‐recurrence survival were identified in colorectal cancer liver metastasis patients. A prognostic nomogram based on these factors was built and validated, which had relatively good discrimination and calibration ability.

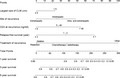

## 1 INTRODUCTION

Colorectal cancer (CRC), the third most common cancer, is the second leading cause of cancer‐associated death globally.^1^ Synchronous or metachronous liver metastases will occur in nearly 50% of CRC patients.^2^ Except for hepatectomy, there has been no other treatment until now offering the possibility of a cure for colorectal cancer liver metastasis (CRLM). Unfortunately, recurrences are detected in more than 50% of CRLM patients within 2 years after the initial resection,^3^ and the 5‐year recurrence possibility is as high as 70%.^3^, ^4^ The high rate of postoperative recurrence remains a major challenge for CRLM patients’ long‐term survival and the 5‐year survival rate is merely 37%–58%.^3^, ^5^, ^6^, ^7^

Multiple prognostic models have been put forward to forecast the postoperative relapse‐free survival (RFS) and overall survival of CRLM patients, and these are primarily built upon the features of the primary tumor and initial liver resection.^8^, ^9^, ^10^ Nevertheless, the effect of prognostic factors at baseline may change dramatically as the disease progresses.^11^, ^12^ For CRLM patients with recurrence, post‐recurrence survival (PRS) may be largely impacted by the characteristics of recurrence and the therapeutic methods for recurrence instead of the characteristics of the primary tumor and initial liver resection.^13^, ^14^, ^15^, ^16^ Constructing effective models to predict PRS is quite warranted. Nomograms are widely regarded as reliable models for individualized prognostic predictions.^17^ By developing an intuitive and user‐friendly graph, nomograms simplify statistical models into a predicted probability for the clinical outcome. In various cancers, they have demonstrated more advantages than traditional staging systems for prognosis prediction.^18^, ^19^, ^20^ However, no well‐established nomogram has been built specifically for personalized PRS prediction of CRLM.

The present study aimed to construct a nomogram to predict PRS integrating the clinical characteristics and treatment methods of recurrence. Therefore, we first established a nomogram for PRS prediction in 251 CRLM patients with post‐hepatectomy recurrence. Then, we validated this prognostic model in an independent cohort of 125 CRLM patients.

## 2 METHODS

### 2.1 Study population

Consecutive CRLM patients who received the first curative hepatectomy during April 2001 and May 2019 at Sun Yat‐sen University Cancer Center (SYSUCC), China, were taken into consideration. Eligibility criteria included: pathologically diagnosed CRLM; both the primary lesions and hepatic metastases underwent radical surgery with tumor‐negative resection margins (R0); and post‐hepatectomy recurrence detected before June 2019. Patients without active follow‐up or adequate clinical information for analysis were excluded, as were patients with a history of other malignancies. The current study was conducted following the Helsinki Declaration. The Medical Ethics Committee of SYSUCC approved this study, with an approval number of B2020‐107–01.

### 2.2 Data acquisition, treatment strategies, and follow‐up

Clinical information of CRLM patients was collected retrospectively from the patients’ medical histories and included general characteristics, primary tumor‐related factors, initial hepatectomy‐related factors, perioperative treatment, and details of recurrence. Postoperative complications of hepatectomy were measured by the FABIB score system.^21^ Comorbidities were documented in the Charlson Comorbidity Index.^22^ The clinical risk score (CRS) ^23^ is the most widely accepted scoring system for predicting CRLM patients' prognosis. The disease‐free interval from primary lesions resection to liver metastasis, the number and the largest diameter of hepatic metastases, and preoperative CEA are four of the criteria that make up the CRS. Therefore, the thresholds of these four variables in the current study were consistent with the CRS system. As for RFS from hepatectomy to recurrence, the largest size of recurrence, and CEA at recurrence, there were no accepted criteria for classification. To optimize the stratification of patients, optimal cutoff values were used. Treatment strategies for CRLM patients mainly referred to the European Society for Medical Oncology guidelines ^24^, ^25^, ^26^ and were discussed by the multidisciplinary team in SYSUCC. Patients were followed until December 2019. Tumor recurrence was confirmed histologically and/or radiographically. PRS was calculated from the date of recurrence to the date of death by any cause.

### 2.3 Statistical analyses

Categorical variables were shown as frequencies and percentages. Continuous variables were presented as the mean±standard deviation (if parametric distribution) or median and interquartile range (if nonparametric distribution). The Chi‐square test or Fisher's exact test was used to compare categorical variables. Optimal cutoff values were calculated by the X‐tile software (version 3.6.1).^27^ Survival proportions were estimated using the Kaplan–Meier (KM) method, with the log‐rank test for comparison. Variables with a *p* value less than 0.1 in the univariable Cox analysis were selected to enter the multivariable Cox regression analysis. The nomogram for PRS was constructed based on the multivariable Cox regression model. Its predictive capacity was measured by Harrell's concordance index (C‐index), KM curves grouped by the tertiles of the nomogram‐predicted score, and calibration plots. The C‐index quantifies the capacity of the nomogram to discriminate patients with different outcomes. It ranges from 0.5 to 1.0, with the minimum value indicating random chance and the maximum value representing perfect discrimination.^28^ The bootstrap resampling for calibration plots was used with 1000 repetitions. Statistical analyses were performed with R software (version 3.5.3; http://www.Rproject.org) or SPSS (Version 23.0; IBM Corp). If not specified, a *p* value below 0.05 was thought to be statistically significant. All *p* values were two‐tailed.

## 3 RESULTS

**FIGURE 1:**
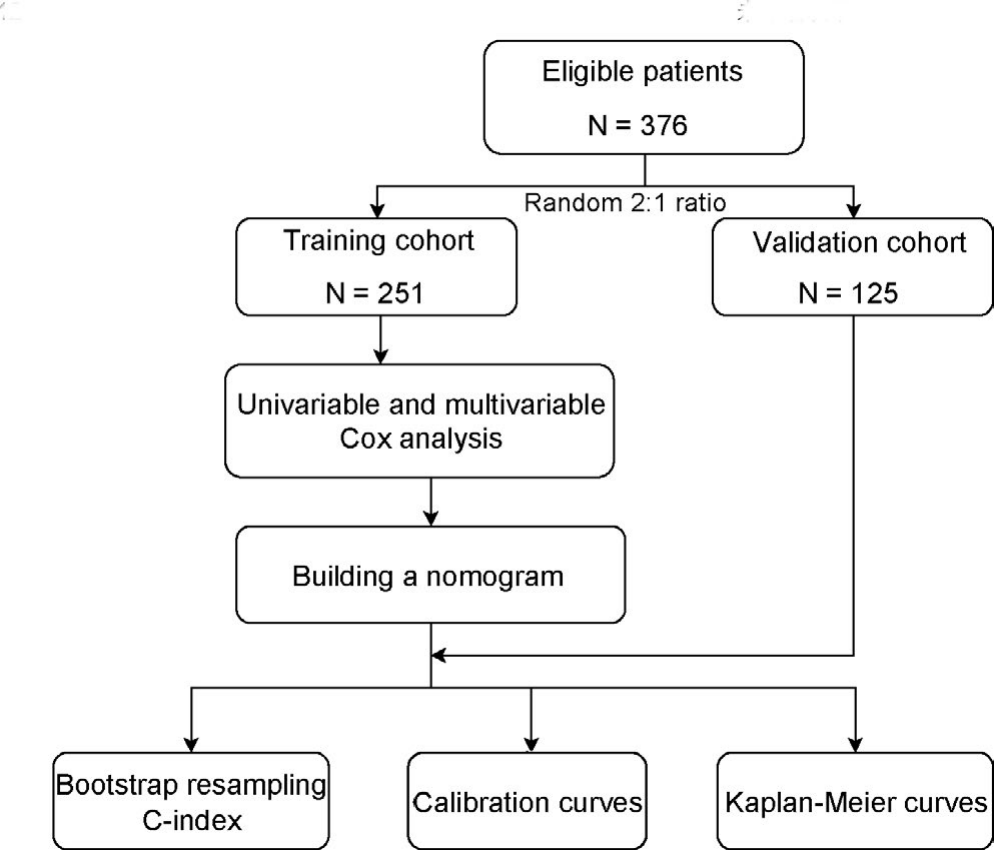
Flowchart of the current study

The flow chart of the current study is presented in Figure 1. About 475 patients in total met the inclusion criteria. Among them, 17 patients (3.6%) with a history of other malignancies, 39 patients (8.2%) without active follow‐up and 43 patients (9.1%) with incomplete recurrence data were excluded. The 3‐year and 5‐year PRS rates of the remaining 376 patients were 37.3% and 24.6%, respectively. Table S1 summarizes the recurrence patterns of the 376 patients. The initial recurrence site was intrahepatic only in 185 (49.2%) patients, lung only in 44 (11.7%) patients, other single sites in 21 (5.6%) patients, and more than two sites in 126 (33.5%) patients (Table S1). These 376 patients were randomly assigned to a training (*n* = 251) and a validation cohort (*n* = 125) at a 2:1 ratio. The median follow‐up was 27.7 months for the training cohort and 26.6 months for the validation cohort. Detailed clinical characteristics of these cohorts are summarized in Table 1. The median RFS for the training and validation cohorts were 6.1 and 6.5 months, respectively.

**TABLE 1:** Clinicopathologic characteristics of 376 eligible patients

Variables	Training cohort *N* = 251	Validation cohort *N* = 125
General characteristics		
Age^b^, year	55.4 ± 11.3	54.5 ± 11.7
Gender, male/female (%)	168/83 (66.9/33.1)	85/40 (68.0/32.0)
RAS mutation, yes/no/NA	52/105/94 (20.7/41.8/37.5)	38/53/34 (30.4/42.4/27.2)
BRAF V600E mutation, yes/no/NA	6/94/151 (2.4/37.5/60.2)	3/54/68 (2.4/43.2/54.4)
MMRstatus, dMMR/pMMR/NA	6/137/108 (2.4/54.6/43.0)	3/81/41 (2.4/64.8/32.8)
Charlson Comorbidity Index, 0/≥1	192/59(76.5/23.5)	82/43(65.6/34.4)
Primary tumor		
Location (%)		
Colon/Rectum	158/93 (62.9/37.1)	82/43 (65.6/34.4)
Right‐sided/Left‐sided	59/192 (23.5/76.5)	22/103 (17.6/82.4)
Tumor grade, G1–2/G3	194/57 (77.3/22.7)	99/26 (79.2/20.8)
T‐stage, T3–4/Tis‐2	231/20 (92.0/8.0)	118/7 (94.4/5.6)
N‐stage, N1–2/N0	165/86 (65.7/34.3)	75/50 (60.0/40.0)
Liver metastases^c^		
DFI, >12 months/≤12 months	43/208 (17.1/82.9)	17/108 (13.6/86.4)
Number of CLM, multiple/single	162/89 (64.5/35.5)	91/34 (72.8/27.2)
Largest size of CLM, >5/≤5, cm	37/214 (14.7/85.3)	19/106 (15.2/84.8)
Preoperative CEA, >200/≤200, ng/ml	16/235 (6.4/93.6)	6/119 (4.8/95.2)
Distribution of CLM, bilobar/unilobar	169/82 (67.3/32.7)	93/32 (74.4/25.6)
Concomitant ablation, yes/no	55/196 (21.9/78.1)	35/90 (28.0/72.0)
FABIB score, 0–2/≥3	242/9(96.4/3.6)	123/2(98.4/1.6)
CRS, 3–5/0–2	119/132 (47.4/52.6)	57/68 (45.6/54.4)
Extrahepatic metastases, Yes/no	24/227 (9.6/90.4)	19/106 (15.2/84.8)
Duration of perioperative chemotherapy, months		
≤3	74 (29.5)	34 (27.2)
3–6	85 (33.9)	46 (36.8)
≥6	92 (36.7)	45 (36.0)
Use of biological agents^a^		
None	189 (75.3)	96 (76.8)
Bevacizumab	38 (15.1)	10 (8.0)
Cetuximab	24 (9.6)	19 (15.2)
Recurrence characteristics		
Relapse‐free survival, year		
≤1	196 (78.1)	95 (76.0)
1–2	35 (13.9)	23 (18.4)
≥2	20 (8.0)	7 (5.6)
Site of recurrence		
Intrahepatic only	134 (53.4)	52 (41.6)
Extrahepatic	58 (23.1)	34 (27.2)
Intrahepatic and extrahepatic	59 (23.5)	39 (31.2)
Number of recurrences, multiple/single	174/77 (69.3/30.7)	94/31 (75.2/24.8)
Largest size of recurrence, ≥3/<3, cm	67/184 (26.7/73.3)	30/95 (24.0/76.0)
CEA at recurrence, ng/ml		
<5	105 (41.8)	43 (34.4)
5–40	106 (42.2)	59 (47.2)
>40	40 (15.9)	23 (18.4)
Treatment of recurrence		
Chemotherapy + Radiotherapy^d^	99 (39.4)	55(44.0)
Resection	42 (16.7)	16(12.8)
Ablation^e^	89 (35.5)	38(30.4)
Other^f^	21 (8.4)	16(12.8)

Note

Data are presented as the means ± SDs or n (%).

Abbreviation: CEA, carcinoembryonic antigen; CLM, colorectal liver metastasis; CRS, clinical risk score; DFI, disease‐free interval from primary tumor resection to liver metastases; dMMR, deficient mismatch repair; NA, not available; pMMR, proficient mismatch repair.

^a^ Perioperative period of initial hepatectomy.

^b^ At recurrence.

^c^ At initial hepatectomy.

^d^ Chemotherapy or radiotherapy, or a combination of the two.

^e^ Radiofrequency ablation, cryoablation, microwave ablation or stereotactic ablative body radiotherapy.

^f^ Supportive care, traditional Chinese medicine.

### 3.1 Prognostic variables for PRS in the training cohort

**FIGURE 2:**
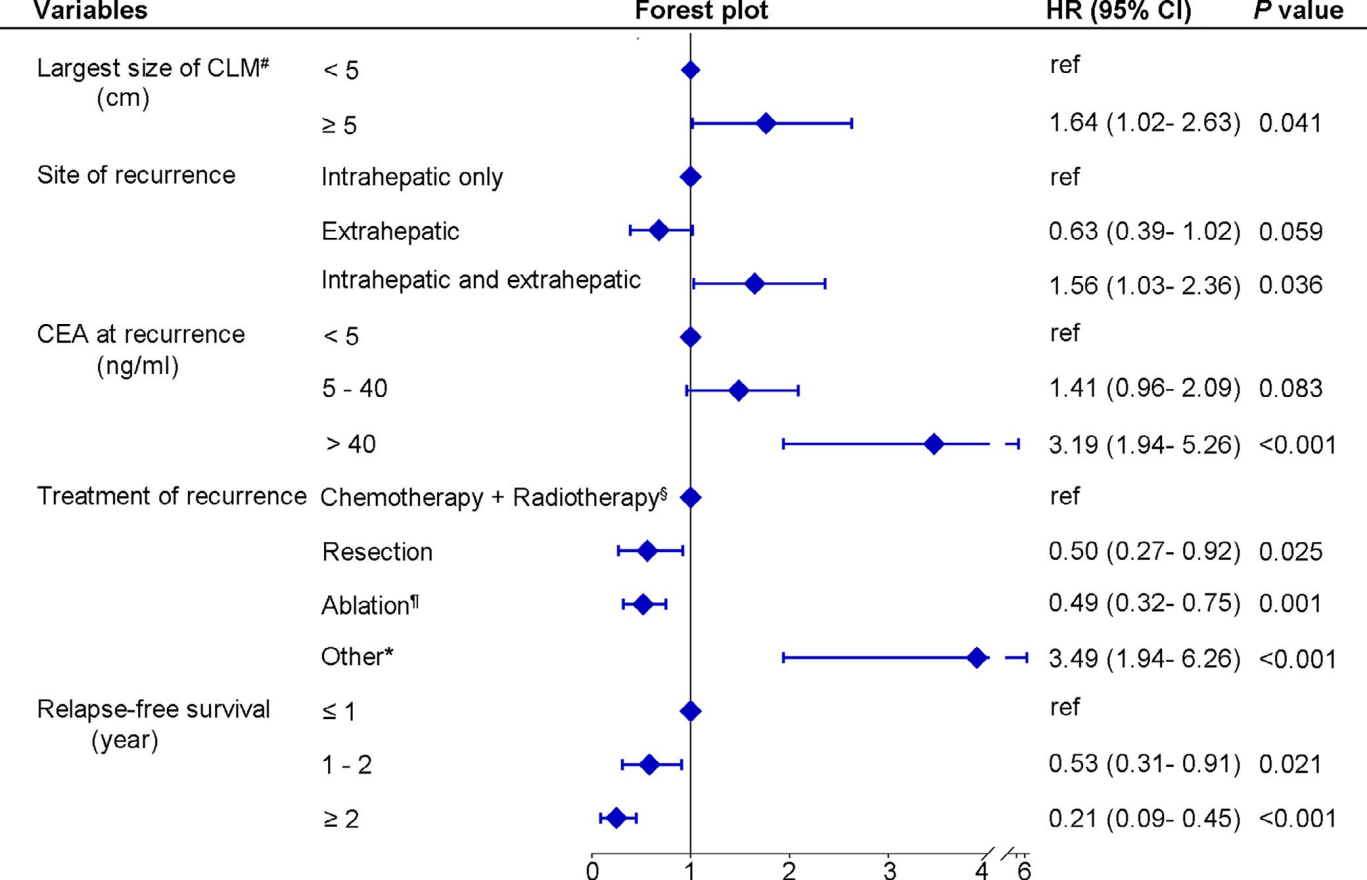
Multivariable Cox analysis and corresponding forest plot of post‐recurrence survival in the training cohort. ^#^At recurrence; ^§^Chemotherapy or radiotherapy, or a combination of the two; ^¶^Radiofrequency ablation, cryoablation, microwave ablation or stereotactic ablative body radiotherapy; ^*^Supportive care, traditional Chinese medicine

Univariable analysis was employed on those 251 patients in the training cohort. The following ten variables with a *p* value below 0.1 were associated with PRS: age at recurrence, pathologic N‐stage, the largest size of hepatic metastases at the time of initial hepatectomy, extrahepatic metastases at the time of initial hepatectomy, RFS from hepatectomy to recurrence, site of recurrence, number of recurrences, the largest size of recurrence, CEA level at recurrence, and treatment for recurrence (Table S2). Multivariable analysis revealed that liver metastases exceeding 5 cm in diameter at the time of initial hepatectomy, intra‐ and extrahepatic recurrence, elevated CEA level at recurrence, and adoption of supportive care or traditional Chinese medicine for recurrence were independently associated with shorter PRS (HR > 1, *p* < 0.05) (Figure 2). In contrast, the adoption of surgical resection or ablation for recurrence and longer RFS from hepatectomy to recurrence were independently associated with prolonged PRS (HR < 1, *p* < 0.05) (Figure 2). KM curves visually demonstrated the differences in PRS among patients grouped by these five independent prognostic factors (Figures S1 and S2).

### 3.2 Construction of a nomogram for predicting PRS

**FIGURE 3:**
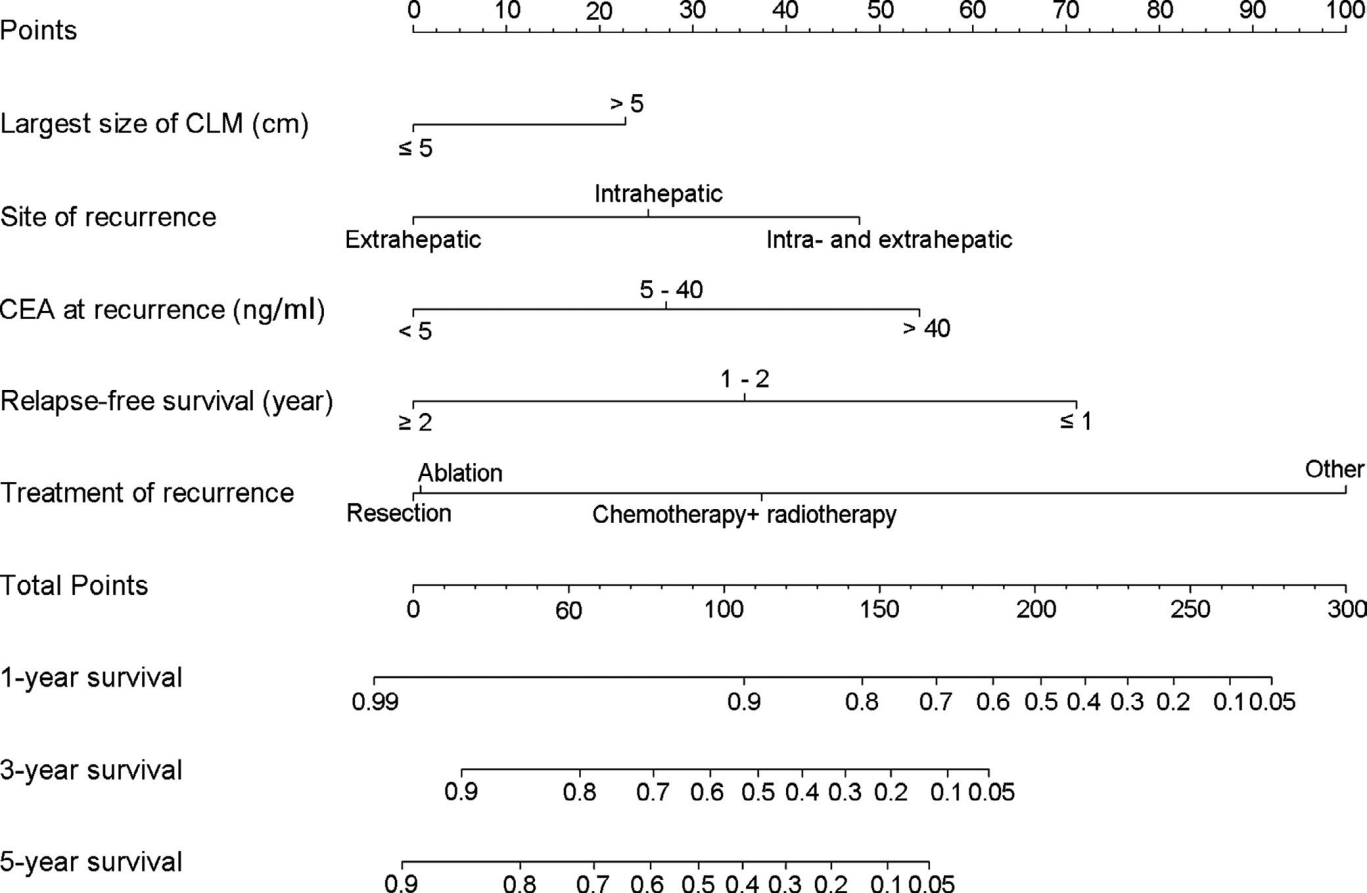
Nomogram for predicting the 1‐, 3‐, and 5‐year post‐recurrence survival rates. Draw a straight line vertically from the axis of each variable to reach the top “Points” scale. Add the score of five variables up then draw a line vertically from the “Total Points” scale to intercept the survival axes. The 1‐, 3‐, and 5‐year post‐recurrence survival rates will be determined accordingly

The five independent prognostic factors above were integrated to construct a prognostic nomogram for PRS (Figure 3). The corresponding score for each variable can be identified by drawing a vertical line upward to the point scale. Get the score for each variable and sum them up. To obtain the estimated rates of 1‐, 3‐, and 5‐year PRS, locate this sum on the “Total Points” axis, and then, draw a line vertically straight down to the survival axes (Figure 3).

### 3.3 Evaluation and validation of the nomogram

**FIGURE 4:**
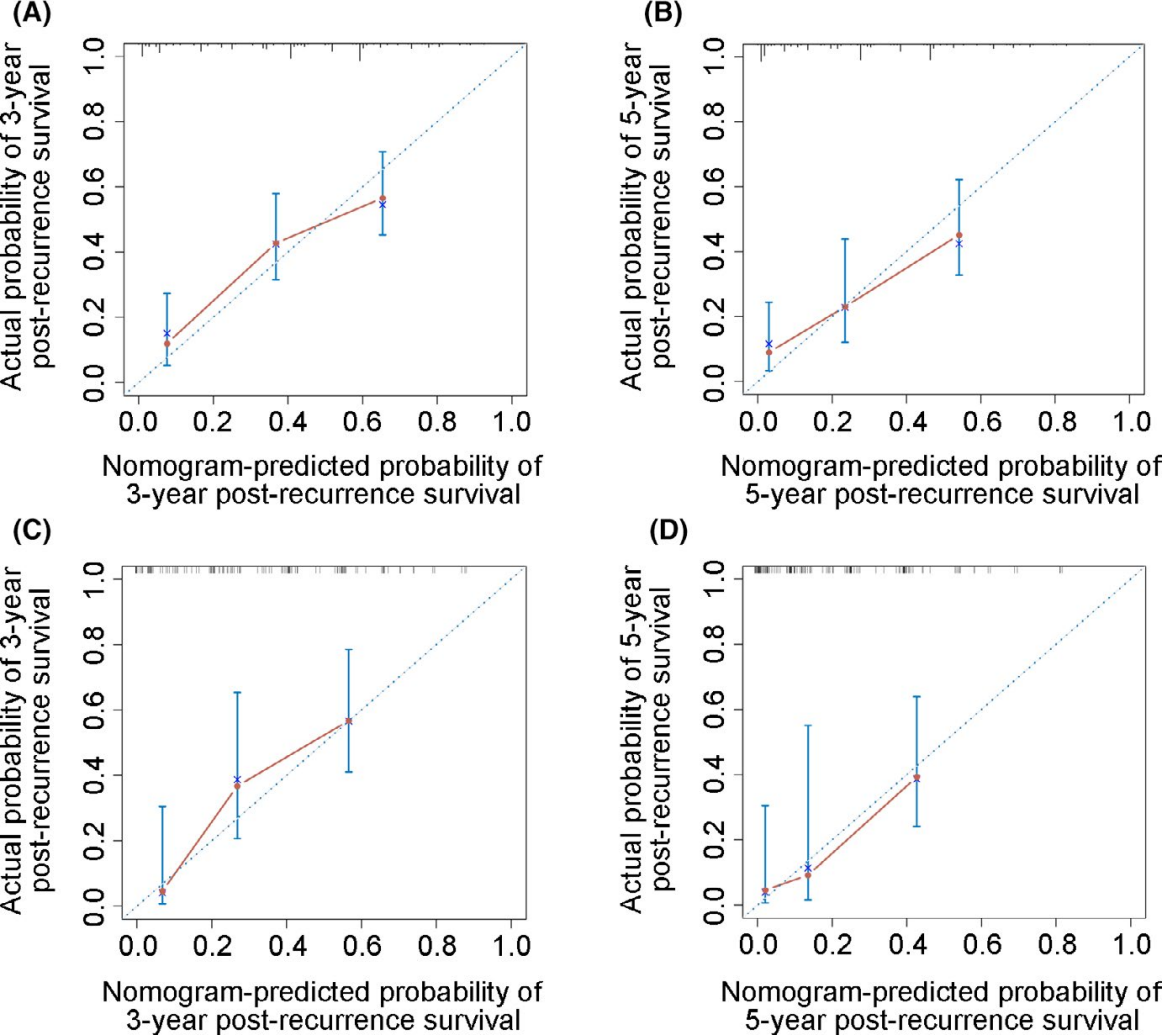
Calibration curves of the nomogram for predicting the 3‐ and 5‐year post‐recurrence survival in the training cohort (A, B) and validation cohort (C, D). The 45° blue dotted line indicates a perfect prediction, and the nomogram's predictive performance is represented by the solid lines. Vertical bars, confidence intervals. The nearest the solid line fitting is to the dotted line, the higher the nomogram's predictive accuracy will be

**FIGURE 5:**
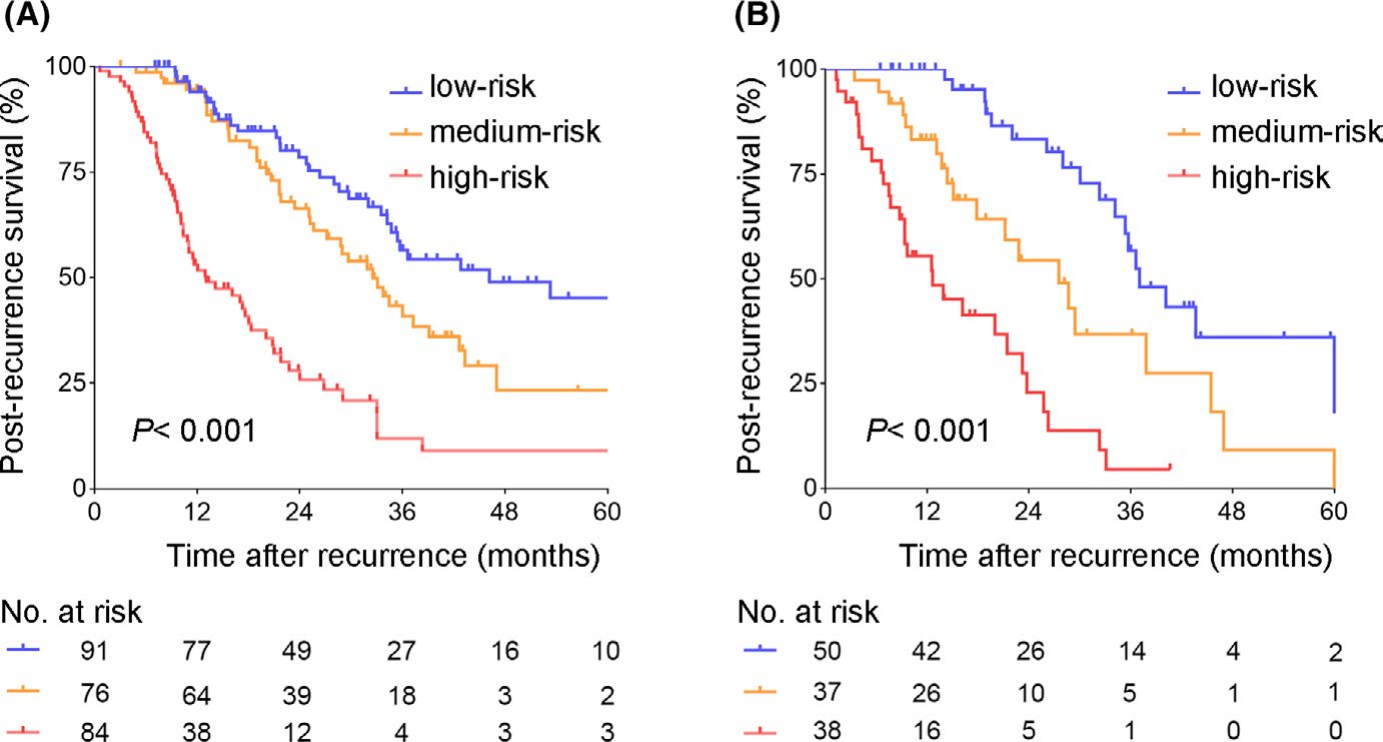
Kaplan–Meier curves for the post‐recurrence survival of CRLM patients from three risk groups. Patients were grouped by the tertile of the nomogram‐predicted score in the training (A) and validation cohort (B). The log‐rank test was performed between groups

With a C‐index value of 0.742, the nomogram had a good discrimination capability in the training cohort. Calibration curves demonstrated that the predicted probability of 3‐year PRS (Figure 4a), and 5‐year PRS (Figure 4b) closely approximated the actual observation. For the validation cohort, a C‐index of 0.773 for PRS prediction was obtained. The nomogram was also well‐calibrated in the validation cohort regardless of the probability of 3‐year PRS (Figure 4c) or 5‐year PRS (Figure 4d). Furthermore, survival curves were used to evaluate the nomogram's discrimination power in predicting PRS (Figure 5a,b). According to the tertile of the model‐predicted score, patients were grouped into low‐risk, medium‐risk, and high‐risk groups with 3‐year PRS rates of 56.6%, 43.2%, and 12.0%, respectively (*p* < 0.001; Figure 5a), and 5‐year PRS rates of 45.2%, 23.3%, and 9.0%, respectively (*p* < 0.001; Figure 5a) in the training cohort. Clinical features of patients in different risk groups are presented in Tables S3 and S4. Different risk groups divided by the tertile were also presented with well‐separated survival curves in the validation cohort (Figure 5b). The 3‐year PRS rates for the low‐, medium‐, and high‐risk subgroups were 56.7%, 36.7%, and 4.6%, respectively (*p* < 0.001; Figure 5b), and the 5‐year PRS rates were 36.0%, 9.2%, and 4.6%, respectively (*p* < 0.001; Figure 5b).

## 4 DISCUSSION

For the first time, a nomogram was constructed and validated to predict the PRS of CRLM patients with post‐hepatectomy recurrence in this study. In clinical practice, the nomogram, an easy‐to‐use scoring tool, enables both physicians and CRLM patients to acquire individualized prediction of PRS.

Previous reports have paid little attention to the PRS of CRLM patients, and the prognostic factors are still ambiguous. In patients with post‐hepatectomy recurrence, only three factors were consistently reported as independent predictors of PRS, including RFS from hepatectomy to relapse, recurrent sites, and therapeutic methods for recurrence.^13^, ^15^, ^16^, ^29^ Our results were consistent with those reported above and confirmed that intra‐ and extrahepatic recurrence was independently associated with shorter PRS, while adoption of surgical resection or ablation for recurrence and longer RFS were favorable prognostic factors. Although early recurrence has been consistently reported to indicate poor PRS, its definition remains ambiguous. Imai et al.^30^ discovered that 8 months after liver resection was the optimal cutoff value of early recurrence, while Liu et al.^10^ defined optimal early recurrences as those occurring within 12 months. In this study, we found that patients' PRS could be better distinguished with 12 months as the cutoff point for early recurrence.

However, several other factors in the literature have also been reported to be correlated with PRS but have not been verified in this study. Such factors include RAS status,^15^ positive lymph node metastasis of the primary tumor,^16^ margins at hepatectomy,^13^ preoperative CEA level,^31^ and disease‐free interval from colectomy to liver metastasis.^13^ Instead, the level of CEA at recurrence, the number of recurrences, and the diameter of recurrent tumors that were not taken into account previously were associated with PRS in this study, although the latter two were not significant predictors in the multivariable analysis. Interestingly, the largest size of liver metastases at the time of initial hepatectomy, a variable commonly used for postoperative prognosis prediction, was also an independent predictor for PRS. Large tumor size may not only increase the difficulty of operation, but may also indicate poor tumor biological behavior.^32^, ^33^

Notably, when recurrence is detected in CRLM patients after the initial hepatectomy, the only prognostic indicator in this nomogram that can intervene is the treatment method for recurrence. In the current study, repeated resection and ablation as treatments for recurrence both led to a considerable survival advantage in terms of median PRS (74.48 and 35.45 months, respectively) compared to that with chemotherapy alone (21.45 months). Favorable survival with repeat resection for CRLM patients has also been reported by previous studies.^34^, ^35^, ^36^, ^37^ Generally, for those patients with better biological behavior, physicians tend to choose aggressive local treatment. In this study, patients who received repeated resection typically had longer RFS (time from the initial hepatectomy to recurrence), fewer instances of intra‐ and extrahepatic recurrence, fewer or smaller tumors at recurrence, and lower levels of CEA at recurrence (Table S5), which was similar to the results of previous reports.^38^ Although these confounding factors were corrected in the multivariable analysis and repeated resection was demonstrated as an independent predictor for PRS, bias toward different treatments may still be influenced by other unknown factors. Nevertheless, considering the outstanding survival advantage with resection, when repeated resection and ablation are both technically feasible, the former is strongly recommended.^39^, ^40^

Our research has several limitations. First, the nomogram was established on the basis of retrospectively collected data from a single institution. Although the nomogram showed good performance in the internal validation cohort, an external cohort is quite necessary to confirm its clinical utility. Second, the genetic status of a significant number of patients was missing in this study. Previous studies have emphasized a close association between molecular characteristics and outcome after CRLM resection.^41^, ^42^, ^43^ Although there was a trend of poor prognosis in patients with RAS‐mutated, BRAF V600E‐mutated, or pMMR tumors, no statistically significant difference was observed in this study (Figure S3). The incorporation of molecular characteristics into the prognostic model may further increase the nomogram's predictive performance.

In conclusion, a prognostic nomogram with five factors may enable physicians to predict individual PRS accurately and easily in CRLM patients with post‐hepatectomy recurrence. Using the nomogram to identify patients at different risk groups of survival might influence the physicians’ decision making.

## CONFLICTS OF INTEREST

There are no potential conflicts of interest.

## AUTHOR CONTRIBUTION

Conception and design: Li YH and Xu RH; Data acquisition: Liu JW, Yuan YF, Li BK, Zheng Y, Wu XJ, Chen G, Wang FH, Wang ZQ, Pan ZZ, and Wan DS; Statistical analysis and interpretation of data: Liang JY, Lin HC, and Wang DS; Drafting the manuscript: Liang JY.

## Supporting information

Fig S1‐3

Table S1

Table S2

Table S3

Table S4

Table S5

## Data Availability

The data should not be publicly available because of ethical concerns.
