# Supplementary material for: A novel prognostic nomogram for colorectal cancer liver metastasis patients with recurrence after hepatectomy
Source: Cancer Med. 2021 Feb 4;10(5):1535–44. doi: 10.1002/cam4.3697 (PMC7940234; doi:10.1002/cam4.3697)
Supplement: Supplementary file 1 — Fig S1‐3 [file CAM4-10-1535-s003.pdf]

Supplemental Figures

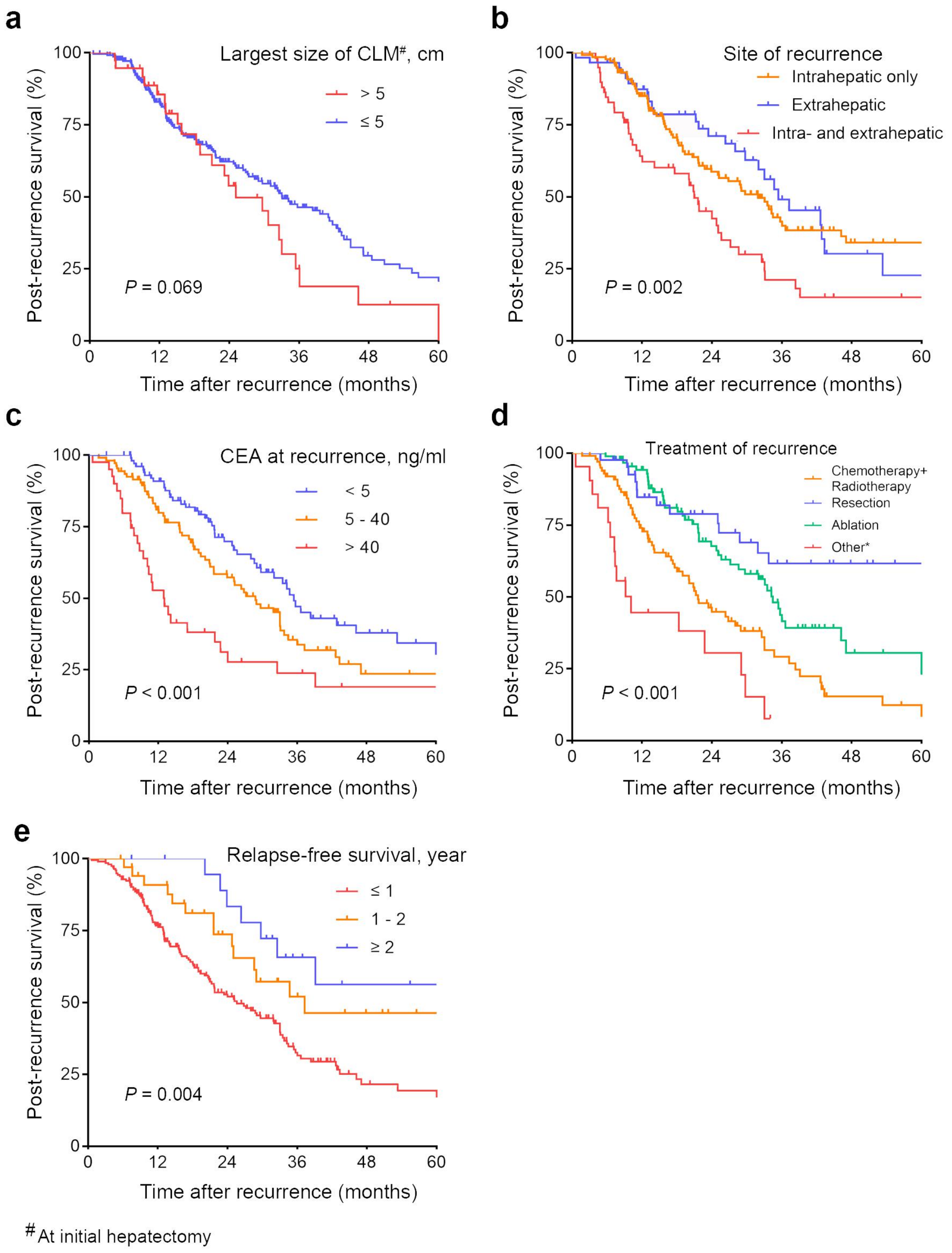

Fig. S1. Kaplan-Meier curves for post-recurrence survival of patients grouped by the five independent prognostic factors in the training cohort.

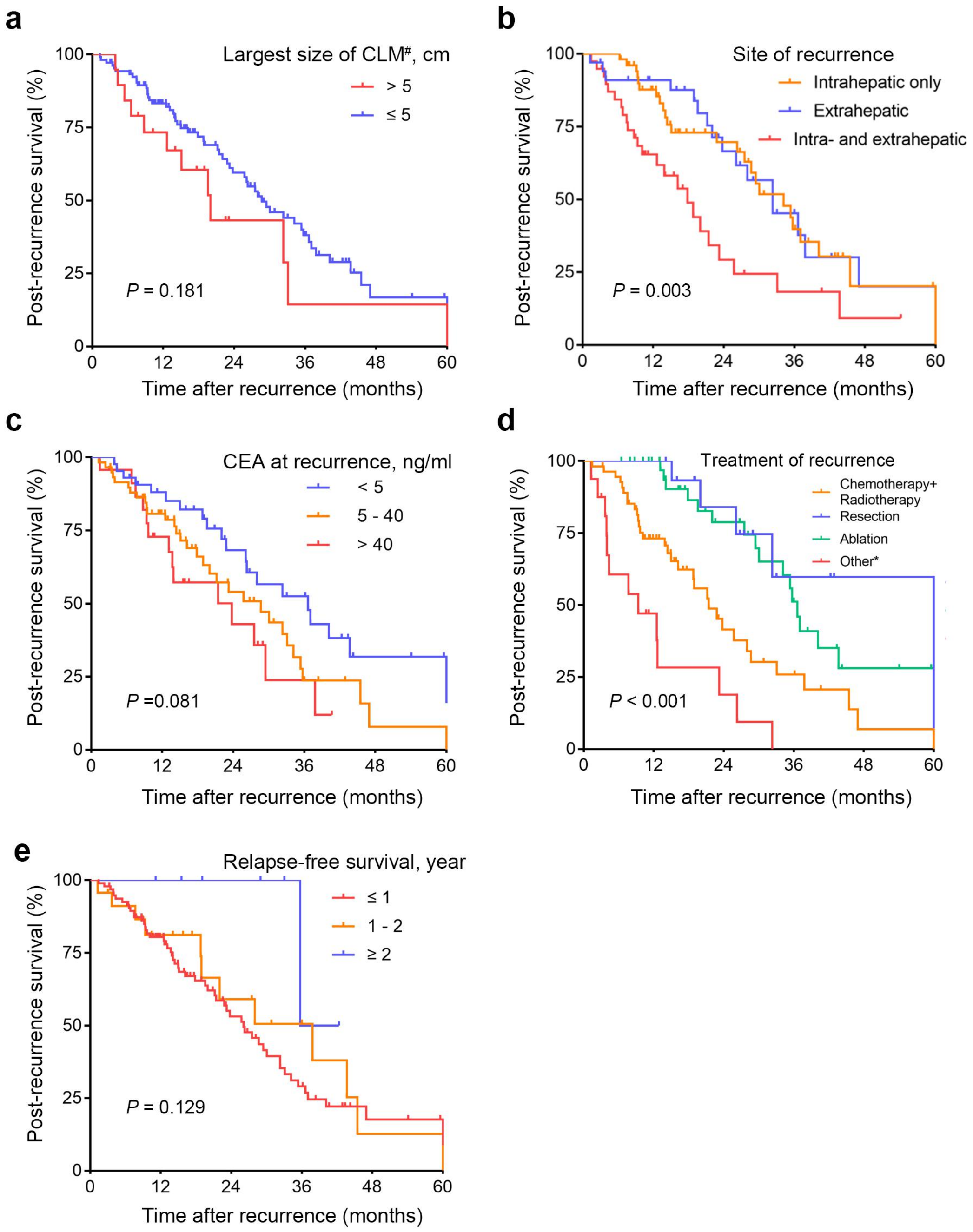

#At initial hepatectomy

Fig. S2. Kaplan-Meier curves for post-recurrence survival of patients grouped by the five independent prognostic factors in the validation cohort.

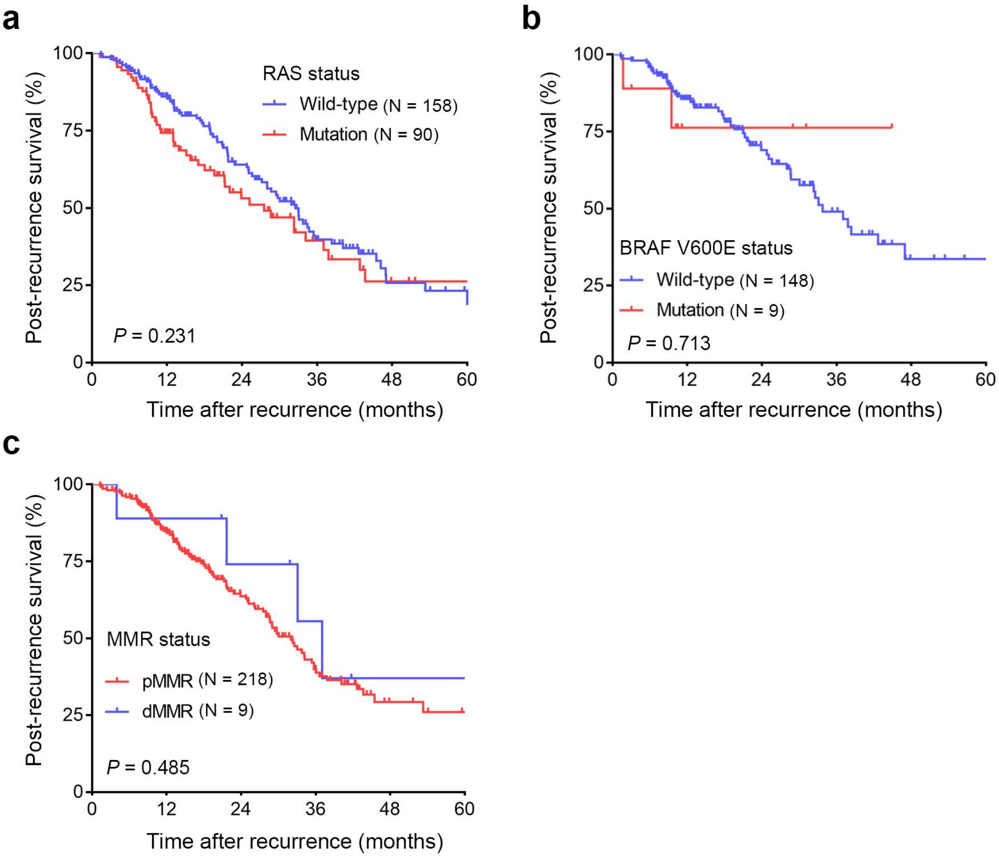

Fig. S3. Kaplan-Meier curves for post-recurrence survival of patients according to the genetic state of RAS (a), BRAF V600E (b) and mismatch repair (MMR, c) in the entire cohort.
