# Supplementary material for: A novel prognostic nomogram for colorectal cancer liver metastasis patients with recurrence after hepatectomy
Source: Cancer Med. 2021 Feb 4;10(5):1535–44. doi: 10.1002/cam4.3697 (PMC7940234; doi:10.1002/cam4.3697)
Supplement: Supplementary file 2 — Table S1 [file CAM4-10-1535-s005.docx]

| **Table S1. Recurrence patterns of all the patients in this study** | |
| --- | --- |
| **Recurrence organ** | **Total (N = 376)** |
| **One organ only** | **66.5%** |
| Intrahepatic only | 49.2% |
| Pulmonary only | 11.7% |
| Primary tumor local recurrence only | 0.3% |
| Peritoneal only | 0.5% |
| Distant lymph nodes only | 2.4% |
| Other one organ#only | 2.4% |
| **Two organs** | **25.3%** |
| Intrahepatic and pulmonary only | 11.2% |
| Intrahepatic and 1 other only | 8.0% |
| Pulmonary and 1 other only | 3.7% |
| Distant lymph nodes and 1 other only | 1.6% |
| Other two organs only | 0.8% |
| **Multi organ (> 2)** | **8.2%** |
| **Liver all** | **75.5%** |
| **Lung all** | **31.6%** |
| #bone/uterus/ovarian/adrenal gland/soft tissue |  |
