# Supplementary material for: A novel prognostic nomogram for colorectal cancer liver metastasis patients with recurrence after hepatectomy
Source: Cancer Med. 2021 Feb 4;10(5):1535–44. doi: 10.1002/cam4.3697 (PMC7940234; doi:10.1002/cam4.3697)
Supplement: Supplementary file 3 — Table S2 [file CAM4-10-1535-s006.docx]

| **Table S2. Univariable COX analysis of post-recurrence survival in the training cohort** | | | | |
| --- | --- | --- | --- | --- |
| **Variables** | **HR** | **HR. 95L** | **HR. 95H** | ***P*** value |
| Agea, year |  |  |  |  |
| ≥65 vs. <65 | 1.72 | 1.15 | 2.57 | **0.009** |
| Gender |  |  |  |  |
| Male vs. female | 1.10 | 0.77 | 1.58 | 0.589 |
| RAS mutation |  |  |  |  |
| No |  |  |  | ref |
| Yes | 1.21 | 0.75 | 1.94 | 0.439 |
| NA | 1.15 | 0.79 | 1.67 | 0.475 |
| Location (%) |  |  |  |  |
| Right-sided/ Left-sided | 0.97 | 0.64 | 1.45 | 0.868 |
| Tumor grade |  |  |  |  |
| G3 vs.G1-2 | 1.24 | 0.83 | 1.86 | 0.292 |
| T-stage |  |  |  |  |
| T3–4 vs. Tis-2 | 0.62 | 0.35 | 1.11 | 0.108 |
| N-stage |  |  |  |  |
| N1–2 vs. N0 | 1.53 | 1.05 | 2.23 | **0.029** |
| Charlson Comorbidity Index |  |  |  |  |
| ≥1 vs. 0 | 0.84 | 0.56 | 1.26 | 0.410 |
| Metastases presentationb |  |  |  |  |
| DFI >12 vs. ≤12 months | 0.85 | 0.55 | 1.30 | 0.443 |
| Number of CLMb |  |  |  |  |
| > 1 vs. ≤ 1 | 1.06 | 0.75 | 1.52 | 0.738 |
| Largest size of CLMb, cm |  |  |  |  |
| > 5 vs. ≤ 5 | 1.51 | 0.97 | 2.38 | **0.071** |
| Preoperative CEAb, ng/ml |  |  |  |  |
| >200 vs. ≤200 | 1.11 | 0.56 | 2.19 | 0.762 |
| Distribution of CLMb |  |  |  |  |
| Bilobar vs.Unilobar | 0.85 | 0.60 | 1.21 | 0.366 |
| Concomitant ablationb |  |  |  |  |
| Yes vs. No | 1.27 | 0.85 | 1.90 | 0.253 |
| FABIB Score |  |  |  |  |
| ≥3 vs. 0-2 | 0.94 | 0.38 | 2.29 | 0.887 |
| Extrahepatic metastasesa |  |  |  |  |
| Yes vs. No | 1.65 | 0.93 | 2.94 | **0.088** |
| Duration of perioperative chemotherapy, months |  |  |  |  |
| ≤3 |  |  |  | ref |
| 3 - 6 | 0.96 | 0.62 | 1.47 | 0.833 |
| ≥6 | 0.97 | 0.64 | 1.45 | 0.876 |
| Use of biological agents# |  |  |  |  |
| None |  |  |  | ref |
| Bevacizumab | 1.17 | 0.73 | 1.88 | 0.509 |
| Cetuximab | 1.03 | 0.60 | 1.77 | 0.922 |
| Relapse-free survival, year |  |  |  |  |
| ≤1 |  |  |  | ref |
| 1-2 | 0.58 | 0.35 | 0.99 | **0.045** |
| ≥2 | 0.37 | 0.18 | 0.77 | **0.007** |
| Site of recurrence |  |  |  |  |
| Intrahepatic only |  |  |  | ref |
| Extrahepatic | 0.89 | 0.57 | 1.39 | 0.609 |
| Intra- and extrahepatic | 1.87 | 1.26 | 2.78 | **0.002** |
| Number of recurrence |  |  |  |  |
| Multiple vs. Single | 2.34 | 1.52 | 3.60 | **<0.001** |
| Largest size of recurrence, cm |  |  |  |  |
| ≥3 vs. <3 | 1.50 | 1.04 | 2.16 | **0.030** |
| CEA at recurrence, ng/ml |  |  |  |  |
| <5 |  |  |  | ref |
| 5 - 40 | 1.49 | 1.02 | 2.19 | **0.042** |
| >40.0 | 2.69 | 1.69 | 4.29 | **<0.001** |
| Treatment for recurrence |  |  |  |  |
| Chemotherapy+ Radiotherapy^§^ |  |  |  | ref |
| Resection | 0.32 | 0.18 | 0.57 | **<0.001** |
| Ablation^¶^ | 0.55 | 0.37 | 0.81 | **0.003** |
| Other* | 2.16 | 1.24 | 3.76 | **0.007** |

Abbreviation: NA, not availble; DFI, disease free interval from primary tumor resection to liver metastases; CEA, carcinoembryonic antigen; CLM, colorectal liver metastasis;CRS，clinical risk score

^#^Perioperative period of initial hepatectomy; ^a^At recurrence; ^b^At initial hepatectomy; ^§^Chemotherapy or radiotherapy, or a combination of the two; ^¶^Radiofrequency ablation, cryoablation, microwave ablation or stereotactic ablative body radiotherapy; ^*^ Supportive care, traditional Chinese medicine

The *P* value in bold indicate statistically significant.
