# Supplementary material for: A novel prognostic nomogram for colorectal cancer liver metastasis patients with recurrence after hepatectomy
Source: Cancer Med. 2021 Feb 4;10(5):1535–44. doi: 10.1002/cam4.3697 (PMC7940234; doi:10.1002/cam4.3697)
Supplement: Supplementary file 4 — Table S3 [file CAM4-10-1535-s001.docx]

| **Table S3. Clinical characteristics of patients in three risk groups stratified by the tertile of nomogram-predicted score in the training cohort** | | | |
| --- | --- | --- | --- |
|  |  |  |  |
| **Variables** | **low-risk**  **N= 91** | **medium-risk**  **N= 76** | **high-risk**  **N= 84** |
| General characteristics |  |  |  |
| Agea, year | 55.0 ± 9.9 | 56.0 ± 12.7 | 58.3 ± 11.3 |
| Gender, Male/Female(%) | 52/39 (57.1/41.9) | 57/19 (75.0/25.0) | 59/25 (70.2/29.8) |
| RAS mutation,Yes/No/NA | 21/36/34(23.1/39.6/37.4) | 13/38/25(17.1/50.0/32.9) | 18/31/35(21.4/36.9/41.7) |
| BRAF V600E mutation, Yes/No/NA | 2/32/57(2.2/35.2/62.6) | 3/32/41 (3.9/42.1/53.9) | 1/30/53 (1.2/35.7/63.1) |
| MMRstatus, dMMR/pMMR/NA | 2/58/31 (2.2/63.7/34.1) | 3/40/33 (3.9/52.6/43.4) | 1/39/44 (1.2/46.4/52.4) |
| Primary tumour |  |  |  |
| Location (%) |  |  |  |
| Colon/Rectum | 58/33 (63.7/36.3) | 45/31 (59.2/40.8) | 55/29 (65.5/34.5) |
| Right-sided/Left-sided | 27/64 (29.7/70.3) | 13/63 (17.1/82.9) | 19/65 (22.6/77.4) |
| Tumor grade, G1-2/G3 | 78/13 (85.7/14.3) | 56/20 (73.7/26.3) | 60/24 (71.4/28.6) |
| Pathological T-stage, T3-4/Tis-2 | 86/5 (94.5/5.5) | 68/8 (89.5/10.5) | 77/7 (91.7/8.3) |
| Pathological N-stage, N1-2/N0 | 55/36 (60.4/39.6) | 45/31 (59.2/40.8) | 65/19 (77.4/22.6) |
| Liver metastasesb |  |  |  |
| DFI, >12months/≤12months | 16/75 (17.1/82.9) | 10/66 (13.2/86.8) | 17/67 (20.2/79.8) |
| Number of CLM, Multiple/Single | 53/38 (58.2/41.8) | 47/29 (61.8/38.2) | 62/22 (73.8/26.2) |
| Largest size of CLM, >5/ ≤5, cm | 4/87 (4.4/95.6) | 13/63 (17.1/82.9) | 20/64 (23.8/76.2) |
| Preoperative CEA, >200/≤200,ng/ml | 2/89 (2.2/97.8) | 7/69 (9.2/90.8) | 7/77 (8.3/91.7) |
| Distribution of CLM, Bilobar/Unilobar | 54/37 (59.3/40.7) | 54/22 (71.1/28.9) | 61/23 (72.6/27.4) |
| Concomitant ablation, Yes/No | 21/70 (23.1/76.9) | 16/60 (21.1/78.9) | 18/66 (21.4/78.6) |
| CRS, 3-5/0-2 | 33/58 (36.3/63.7) | 35/41 (46.1/53.9) | 51/33 (60.7/39.3) |
| Extrahepatic metastases,Yes/No | 10/81 (11.0/89.0) | 4/72 (5.3/94.7) | 10/74 (11.9/88.1) |
| Duration of perioperative chemotherapy, months |  |  |  |
| ≤3 | 25 (27.5) | 22 (28.9) | 27 (32.1) |
| 3 - 6 | 30 (33.) | 20 (26.3) | 35 (41.7) |
| ≥6 | 36 (39.6) | 34 (44.7) | 22 (26.2) |
| Use of biological agents# |  |  |  |
| None | 66 (72.5) | 58 (76.3) | 65 (77.4) |
| Bevacizumab | 16 (17.6) | 12 (15.8) | 10 11.9) |
| Cetuximab | 9 (9.9) | 6 (7.9) | 9 (10.7) |
| Recurrence characteristics |  |  |  |
| Relapse-free survival,year |  |  |  |
| ≤1 | 56 (61.5) | 61 (80.3) | 79 (94.0) |
| 1-2 | 20 (22.0) | 11 (14.5) | 4 (4.8) |
| ≥2 | 15 (16.5) | 4 (5.3) | 1 (1.2) |
| Recurrence site |  |  |  |
| Intrahepatic only | 55 (60.4) | 42 (55.3) | 37 (44.0) |
| Extrahepatic | 34 (37.4) | 14 (18.4) | 10 (11.9) |
| Intrahepatic and extrahepatic | 2 (2.2) | 20 (26.3) | 37 (44.0) |
| Number of recurrence, Multiple/Single | 47/44 (51.6/48.4) | 54/22 (71.1/28.9) | 73/11 (86.9/13.1) |
| Largest size of recurrence, ≥3 / <3, cm | 19/72 (20.9/79.1) | 15/61 (19.7/80.3) | 33/51 (39.3/60.7) |
| CEA at recurrence, ng/ml |  |  |  |
| < 5 | 72 (79.1) | 21 (27.6) | 12 (14.3) |
| 5 - 40 | 16 (17.6) | 51 (67.1) | 39 (46.4) |
| > 40 | 3 (3.3) | 4 (5.3) | 33 (39.3) |
| Treatment of recurrence |  |  |  |
| Chemotherapy+Radiotherapy^§^ | 16 (17.6) | 28 (36.8) | 55 (65.5) |
| Resection | 31 (34.1) | 9 (11.8) | 2 (2.4) |
| Ablation^¶^ | 43 (47.3) | 38 (50.0) | 8 (9.5) |
| Other* | 1 (1.1) | 1 (1.3) | 19 (22.6) |

Abbreviation: NA, not availble; DFI, disease free interval from primary tumor resection to liver metastases; CEA, carcinoembryonic antigen; CLM, colorectal liver metastasis;CRS，clinical risk score

^#^Perioperative period of initial hepatectomy; ^a^At recurrence; ^b^At initial hepatectomy; ^§^Chemotherapy or radiotherapy, or a combination of the two; ^¶^Radiofrequency ablation, cryoablation, microwave ablation or stereotactic ablative body radiotherapy; ^*^ Supportive care, traditional Chinese medicine
