# Supplementary material for: A novel prognostic nomogram for colorectal cancer liver metastasis patients with recurrence after hepatectomy
Source: Cancer Med. 2021 Feb 4;10(5):1535–44. doi: 10.1002/cam4.3697 (PMC7940234; doi:10.1002/cam4.3697)
Supplement: Supplementary file 5 — Table S4 [file CAM4-10-1535-s004.docx]

| **Table S4. Clinical characteristics of patients in three risk groups stratified by the tertile of nomogram-predicted score in the validation cohort** | | | |
| --- | --- | --- | --- |
|  |  |  |  |
| **Variables** | **low-risk**  **N= 50** | **medium-risk**  **N= 37** | **high-risk**  **N= 38** |
| General characteristics |  |  |  |
| Agea, year | 53.5 ± 10.3 | 57.0 ± 10.9 | 57.0 ± 14.3 |
| Gender, Male/Female(%) | 36/14 (72.0/28.0) | 23/14 (62.2/37.8) | 26/12 (68.4/31.6) |
| RAS mutation,Yes/No/NA | 15/19/16 (30.0/39.0/32.0) | 16/17/4 (43.2/45.9/10.8) | 7/17/14 (18.4/44.7/36.8) |
| BRAF V600E mutation, Yes/No/NA | 3/19/28(6.0/38.0/56.0) | 0/22/15 (0.0/59.5/40.5) | 0/13/25 (0/34.2/65.8) |
| MMRstatus, dMMR/pMMR/NA | 2/36/12 (4.0/72.0/24.0) | 0/25/12 (0.0/67.6/32.4) | 1/20/17 (2.6/52.6/44.7) |
| Primary tumour |  |  |  |
| Location (%) |  |  |  |
| Colon/Rectum | 31/18 (62.0/38.0) | 26/11 (70.3/29.7) | 25/13 (65.8/34.2) |
| Right-sided/Left-sided | 6/44 (12.0/88.0) | 9/28 (24.3/75.7) | 7/31 (18.4/81.6) |
| Tumor grade, G1-2/G3 | 43/7 (86.0/14.0) | 30/7 (81.1/18.9) | 26/12 (68.4/31.6) |
| Pathological T-stage, T3-4/Tis-2 | 46/4 (92.0/8.0) | 35/2 (94.6/5.4) | 37/1 (97.4/2.6) |
| Pathological N-stage, N1-2/N0 | 33/17 (66.0/34.0) | 21/16 (56.8/43.2) | 21/17 (55.3/44.7) |
| Liver metastasesb |  |  |  |
| DFI, >12months/≤12months | 9/41 (18.0/82.0) | 2/35 (5.4/94.6) | 6/32 (15.8/84.2) |
| Number of CLM, Multiple/Single | 32/18 (64.0/36.0) | 29/8 (78.4/21.6) | 30/8 (78.9/21.1) |
| Largest size of CLM, >5/ ≤5, cm | 4/46 (8.0/92.0) | 4/33 (10.8/89.2) | 11/27 (28.9/71.1) |
| Preoperative CEA, >200/≤200,ng/ml | 3/47 (6.0/94.0) | 1/36 (2.7/97.3) | 2/36 (5.3/94.7) |
| Distribution of CLM, Bilobar/Unilobar | 32/18 (64.0/36.0) | 30/7 (81.1/18.9) | 31/7 (81.6/18.4) |
| Concomitant ablation, Yes/No | 11/39 (22.0/78.0) | 15/22 (40.5/59.5) | 9/29 (23.7/76.3) |
| CRS, 3-5/0-2 | 20/30 (40.0/60.0) | 17/20 (45.9/54.1) | 20/18 (52.6/47.4) |
| Extrahepatic metastases,Yes/No | 6/44 (12.0/88.0) | 3/34 (8.1/91.9) | 10/28 (26.3/73.7) |
| Duration of perioperative chemotherapy, months |  |  |  |
| ≤3 | 18 (36.0) | 6 (16.2) | 10 (26.3) |
| 3 - 6 | 18 (36.0) | 17 (45.9) | 11 (28.9) |
| ≥6 | 14 (28.0) | 14 (37.8) | 17 (44.7) |
| Use of biological agents# |  |  |  |
| None | 40 (80.0) | 27 (73.0) | 29 (76.3) |
| Bevacizumab | 2 (4.0) | 4 (10.8) | 4 (10.5) |
| Cetuximab | 8 (16.0) | 6 (16.2) | 5 (13.2) |
| Recurrence characteristics |  |  |  |
| Relapse-free survival,year |  |  |  |
| ≤1 | 32 (64.0) | 31 (83.8) | 32 (84.2) |
| 1-2 | 12 (24.0) | 5 (13.5) | 6 (15.8) |
| ≥2 | 6 (12.0) | 1 (2.7) | 0 (0.0) |
| Recurrence site |  |  |  |
| Intrahepatic only | 23 (46.0) | 23 (62.2) | 6 (15.8) |
| Extrahepatic | 22 (44.0) | 7 (18.9) | 5 (13.2) |
| Intrahepatic and extrahepatic | 5 (10.0) | 7 (18.9) | 27 (71.1) |
| Number of recurrence, Multiple/Single | 29/21 (58.0/42.0) | 30/7 (81.1/18.9) | 35/3 (92.1/7.9) |
| Largest size of recurrence, ≥3 / <3, cm | 5/45 (10.0/90.0) | 7/30 (18.9/81.1) | 18/20 (47.4/52.6) |
| CEA at recurrence, ng/ml |  |  |  |
| < 5 | 30 (60.0) | 7 (18.9) | 6 (15.8) |
| 5 - 40 | 19 (38.0) | 21 (56.8) | 19 (50.0) |
| > 40 | 1 (2.0) | 9 (24.3) | 13 (34.2) |
| Treatment of recurrence |  |  |  |
| Chemotherapy+Radiotherapy^§^ | 5 (10.0) | 28 (75.7) | 22 (57.9) |
| Resection | 14 (28.0) | 1 (2.7) | 1 (2.6) |
| Ablation^¶^ | 30 (60.0) | 8 (21.6) | 0 (0.0) |
| Other* | 1 (2.0) | 0 (0.0) | 15 (39.5) |

Abbreviation: NA, not availble; DFI, disease free interval from primary tumor resection to liver metastases; CEA, carcinoembryonic antigen; CLM, colorectal liver metastasis;CRS，clinical risk score

^#^Perioperative period of initial hepatectomy; ^a^At recurrence; ^b^At initial hepatectomy; ^§^Chemotherapy or radiotherapy, or a combination of the two; ^¶^Radiofrequency ablation, cryoablation, microwave ablation or stereotactic ablative body radiotherapy; ^*^ Supportive care, traditional Chinese medicine
