# Supplementary material for: A novel prognostic nomogram for colorectal cancer liver metastasis patients with recurrence after hepatectomy
Source: Cancer Med. 2021 Feb 4;10(5):1535–44. doi: 10.1002/cam4.3697 (PMC7940234; doi:10.1002/cam4.3697)
Supplement: Supplementary file 6 — Table S5 [file CAM4-10-1535-s002.docx]

| **Table S5. Recurrence characteristics of all the** **patients receiving different treatmet for recurrence in this study.** | | | | | |
| --- | --- | --- | --- | --- | --- |
| Variables | Resection | | Ablation | Chemotherapy / Radiotherapy | *P* value |
| **Recurrence characteristics** | N = 58 | | N = 127 | N = 154 |  |
| Relapse-free survival, year |  | |  |  | **< 0.001** |
| ≤1 | 32(55.2) | | 106(83.5) | 126(81.8) |  |
| 1-2 | 13(22.4) | | 17(13.4) | 22(14.3) |  |
| ≥2 | 13(22.4) | | 4(3.1) | 6(3.9) |  |
| Recurrence site |  | |  |  | **< 0.001** |
| Intrahepatic only | 33(56.9) | | 90(70.9) | 51(33.1) |  |
| Extrahepatic | | 20(34.5) | 16(12.6) | 44(28.6) |  |
| Intra- and extrahepatic | | 5(8.6) | 21(16.5) | 59(38.3) |  |
| Number of recurrence | |  |  |  | **< 0.001** |
| Single | | 32(55.2) | 51(40.2) | 20(13.0) |  |
| Multiple | | 26(44.8) | 76(59.8) | 134(87.0) |  |
| Largest size of recurrence, cm | |  |  |  | **< 0.001** |
| <3 | | 36(62.1) | 115(90.6) | 107(69.5) |  |
| ≥3 | | 22(37.9) | 12(9.4) | 47(30.5) |  |
| CEA at recurrence, ng/ml | |  |  |  | **0.003** |
| < 5 | | 30(51.7) | 58(45.7) | 46(29.9) |  |
| 5 - 40 | | 23(39.7) | 54(42.5) | 72(46.8) |  |
| > 40 | | 5(8.6) | 15(11.8) | 36(23.4) |  |
| Data are presented as n (%). | |  |  |  |  |
